# Décollement geometry controls on shallow very low frequency earthquakes

**DOI:** 10.1038/s41598-022-06645-2

**Published:** 2022-02-17

**Authors:** Yoshitaka Hashimoto, Shigeyuki Sato, Gaku Kimura, Masataka Kinoshita, Ayumu Miyakawa, Gregory F. Moore, Masaru Nakano, Kazuya Shiraishi, Yasuhiro Yamada

**Affiliations:** 1 Department of Global Environment and Disaster Prevention, Faculty of Science and Technology, Kochi University, Akebonocho 2-5-1, Kochi, 780-8520 Japan; 2 Japan Agency for Marine-Earth Science and Technology, 3173-25 Showa-machi, Kanazawa-ku, Yokohama, Kanagawa 236-0001 Japan; 3 Earthquake Research Institute, The University of Tokyo, 1-1-1 Yayoi, Bunkyo-ku, Tokyo, 113-0032 Japan; 4 Geological Survey of Japan, National Institute of Advanced Industrial Science and Technology, 1-1-1 Higashi, Tsukuba City, Ibaraki 305-8567 Japan; 5 Department of Earth Sciences, University of Hawai’i, Mānoa, 1680 East-West Road, Honolulu, HI 96822 USA; 6 Department of Earth Resources Engineering, Graduate School of Engineering, Kyushu University, Motooka 774, Fukuoka, 819-0395 Japan

**Keywords:** Structural geology, Seismology

## Abstract

Recent studies have documented the occurrence of shallow very low frequency earthquakes (VLFE) in subduction zones. The heterogeneity of the materials or stresses that act on the plate interface results in the variable slip rate. Stress on the décollement can be controlled by the décollement geometry and the regional stress, which is also able to control the material properties. We determined the distribution of stress along the shallow portion of the décollement in the Nankai Trough using a three-dimensional (3D) seismic survey and regional stress analysis to construct maps of normalized slip tendency (*T*_*s*_′) and dilation tendency (*T*_*d*_). Alignments of VLFEs trend parallel to the trends of ${T}_{s}^{^{\prime}}$ and ${T}_{d}$. On the other hand, very low ${T}_{s}^{^{\prime}}$ and ${T}_{d}$ areas probably act as barriers that limit the number of VLFEs that can migrate towards the trench. Because the ${T}_{s}^{^{\prime}}$ and ${T}_{d}$ distributions are derived only from the décollement geometry and the regional stress without incorporating any data on sediment properties, the consistency between the trends suggests that the décollement geometry is the primary control on VLFE activity.

## Introduction

Faults that slip with varying velocities, including slow and fast earthquakes, are commonly observed along circum-Pacific subduction zones^1^. Tsunamigenic earthquakes are produced by fast-slip earthquakes in the shallow region of the subducting plate interfaces^2,3^. Recently, the number of reports of slow earthquakes including slow-slip events (SSE), tremor, and very low frequency earthquakes (VLFE), in shallow subduction zones has increased^4,5^, and those studies show that slow earthquakes also occur in the same areas where tsunamigenic-fast earthquakes are observed.

A conceptual model for variable slip has been proposed, suggesting that small unstable patches surrounded by a ductile matrix along the plate interface can result in complex seismic energy release^6^. This model is supported by numerical experiments showing that rheological heterogeneity along the plate interface explains slip variability^7^. The model also suggests that slow slip events can trigger tremors or VLFE. It is possible that rheological contrasts could be related to stress heterogeneities, which are controlled not only by the physical properties of the rocks, but also by the geometry of the plate boundary^8,9^. A two-dimensional model used to examine the effects of subducting plate topography, including seamounts, showed the heterogeneous distribution of physical properties and stress around the features^10^. However, stress heterogeneities related to décollement geometry have not yet been well examined in a natural setting, except for a recent study in Costa Rica which examined the qualitative effect of décollement geometry on slip behaviors^11^.

**Figure 1:**
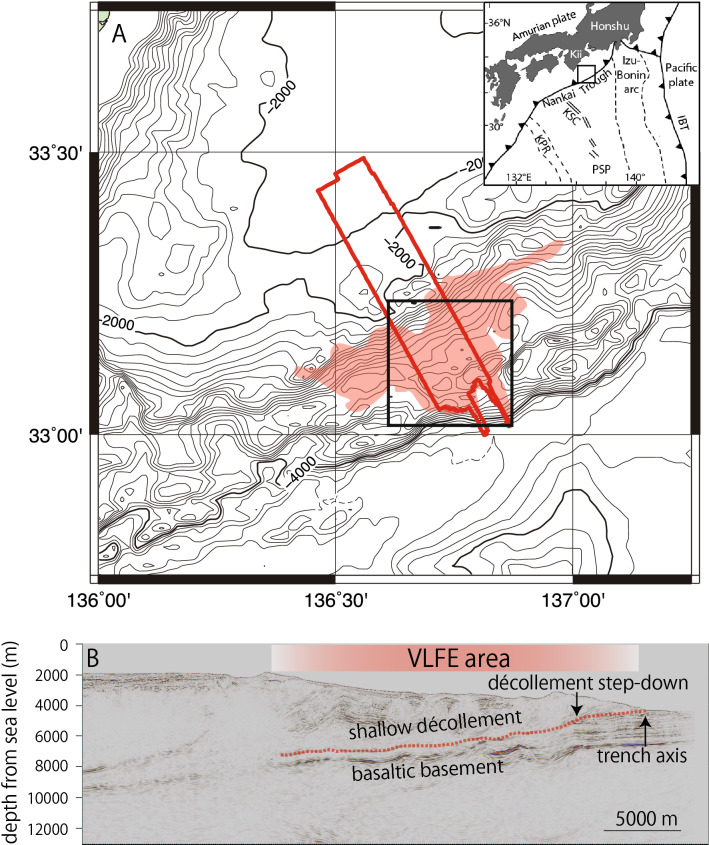
(**A**) Study area. The location of (**A**) is indicated by the box in the regional map in the upper right inset (*IBT* Izu-Bonin Trench, *Kii* Kii Peninsula, *KSC* Kinan seamount chain, *PSP* Philippine Sea Plate, *KPR* Kyushu-Palau Ridge). The red line indicates the 3D seismic survey area. The black rectangle indicates the area shown in Figs. 2 and 3. The distribution of a series of the shallow VLFE events in April 2016 is represented by the red area. (**B**) A seismic in-line section from the 3D seismic survey. The red dashed line indicates the extent of the shallow décollement examined in this study. The shallow VLFE zone is represented by a red area.

In this study, we derive the stress distribution along a subducting plate interface from the relationship between the 3D geometry of the décollement and the regional stress. The study area is the Nankai Trough, located off the Kii Peninsula in Japan, for which a 3D seismic survey is available^12^ (Fig. 1). Tectonic tremor, VLFEs, and SSEs were recorded in the area of the 3D seismic survey^5,13^ by an ocean bottom observation network (Dense Ocean floor Network system for Earthquakes and Tsunamis, DONET). An advantage of our study is that the regional stress in the study area can be estimated from the Centroid–Moment–Tensor (CMT) mechanisms of the VLFE, which can then be used to examine normalized slip tendency (${T}_{s}^{^{\prime}}$) and dilation tendencies (${T}_{d}$) as a function of stress along the uneven décollement. Finally, we compare map views of ${T}_{s}^{^{\prime}}$ and ${T}_{d}$ with the distribution of VLFE to discuss the effect of the décollement geometry on the occurrence of VLFE in the Nankai Trough.

The Nankai Trough is a convergent plate boundary located along southwest Japan, where the Philippine Sea Plate subducts beneath the Amurian Plate^14^, with a convergence rate of 4–6.5 cm/year (~ 5.8 cm/year off the Kii Peninsula) at an azimuth of ~ 300°–315°^15,16^. The 3D seismic survey data were collected in this area to characterize the internal structures and elastic properties of the accretionary prism prior to operations by the Integrated Ocean Drilling Program (IODP) as part of the Nankai Trough Seismogenic Zone Experiment (NanTroSEIZE)^12,17,18^. We use a series of VLFE excitations recorded during April 1–17, 2016 in the region of the 3D seismic survey. More than 300 VLFE were identified in the series and all these events were analyzed to generate CMT mechanisms using DONET^5^. Those mechanisms were then used for regional stress estimation.

## Results

### 3D surface of the shallow décollement

**Figure 2:**
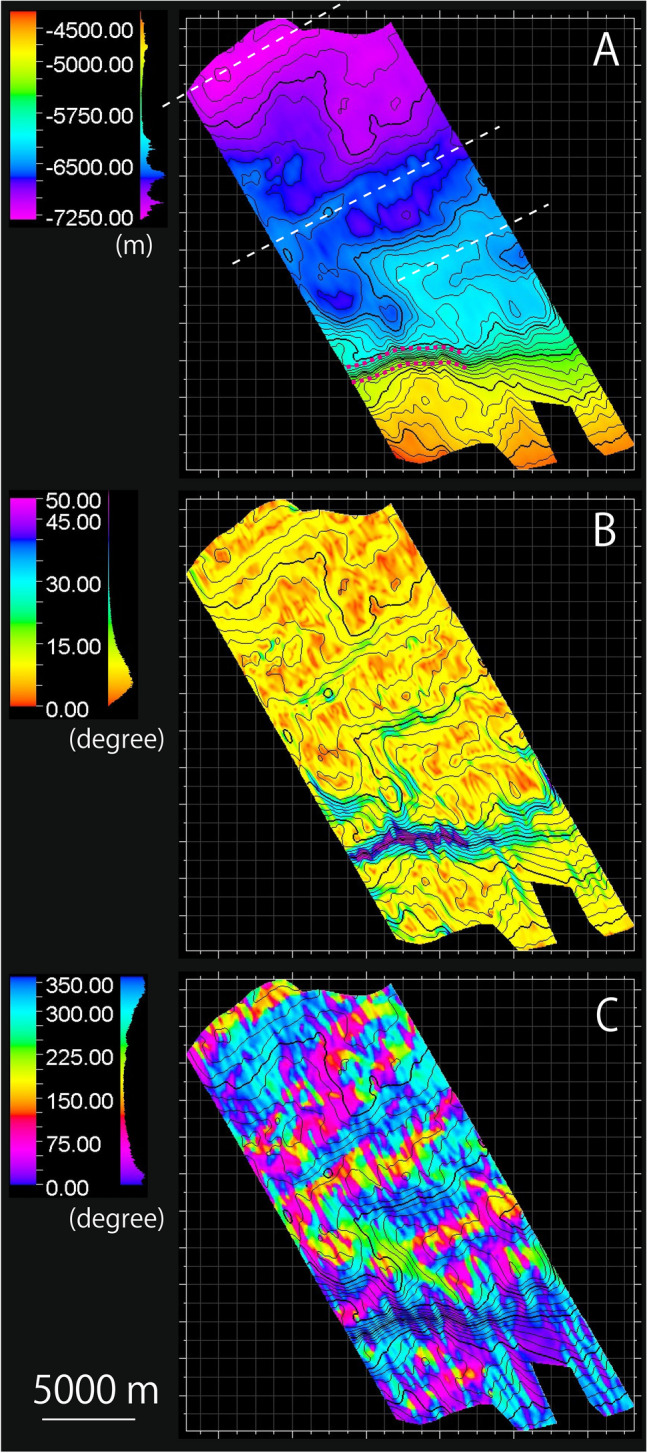
(**A**) Depth map of the décollement in meters below sea level. White dashed lines indicate axes of uplift or subsidence on the décollement. Red dashed lines outline the zone of steep dips. (**B**) Average dip for each 50 m × 50 m mesh surface. (**C**) Average dip azimuth for each 50 m × 50 m mesh surface. The dip azimuth is measured clockwise from North. Color bars and histograms (the number of mesh surfaces with the values) of depth, dip, and dip azimuth are shown to the left of each image.

The depth below sea level of the shallow décollement ranged from 4250 to 7350 m (Fig. 2A). The depth generally increased from SE to NW, almost parallel to the direction of plate convergence. Local uplift and depression of the décollement were observed (Fig. 2A), approximately parallel to the structural trend within the basaltic basement (Fig. 1B). After dividing the décollement into 50 m × 50 m mesh surfaces, dip angle (plunge) and dip azimuth (dip direction on a horizontal surface, represented by clockwise angle from north) of each surface were extracted. The dip is shown as the absolute value without any information of dip orientation (Fig. 2B). The dip ranged mainly from 0° to 15° while the dip azimuth was 0°–100° or 300°–360° (Fig. 2B,C). The variations in dip and dip azimuth were weakly distributed in the NE–SW or ENE–WSW directions. Steeply-dipping areas (45°–50°) were identified in the southern region of the décollement, likely related to a décollement step-down in the shallow portion of the décollement (Figs. 1B, 2A,B).

### Regional stress from VLFE slip data and the distributions of slip and dilation tendencies

We estimated the dip azimuth and dip angle of the optimal solution using the stress inversion method (see “Methods” section), for the maximum, intermediate, and minimum principal stresses, which were (139°, 45°), (234°, 5°), and (329°, 45°) (Fig. S1). The stress ratio of the optimal solution was determined to be 0.76. The variations of the dip azimuth, dip angle and stress ratio of the maximum principal stresses calculated at a slightly smaller number of inverted solutions for the normalized frequency, 0.95 of *F*^*(i)*^, ranges ~  ± 30°, ~  − 30° and ~  − 0.5 (Fig. S1A). We named the stress states with the variation around the optimal solution as the “preferred stress state”. The stress inversion method can identify multiple stress states as multiple clusters of solutions if multiple stress states exist in time and space. Although the inverted stress state has a range of variations, the estimated stress state, including both the optimal solution and preferred stress state, was well defined as a single cluster for the maximum principal stress (dip azimuth of 110°–120°). Because the distribution of VLFE in this area was sufficiently widespread to cover the 3D seismic survey area, the single cluster of stress orientations can be interpreted as the regional stress for the entire area.

**Figure 3:**
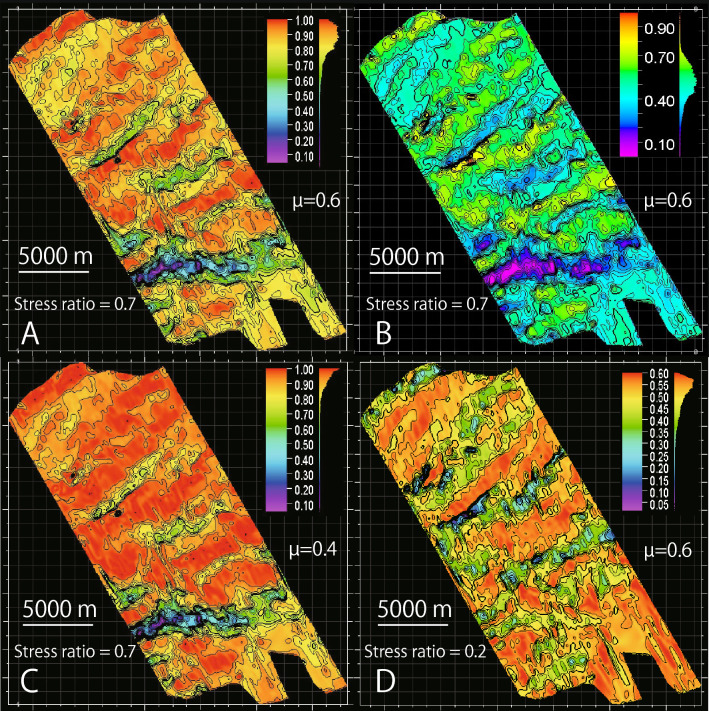
(**A**) Distribution of ${T}_{s}^{^{\prime}}$ with a friction coefficient of 0.6 and the optimal regional stress state. (**B**) Distribution of ${T}_{d}$ with a friction coefficient of 0.6 and the optimal regional stress state. (**C**) ${T}_{s}^{^{\prime}}$ with a friction coefficient of 0.4 and the optimal regional stress state. (**D**) Distribution of ${T}_{s}^{^{\prime}}$ with a friction coefficient of 0.6 and preferred regional stress state (the horizontal maximum stress and 0.2 of stress ratio).

Slip and dilation tendencies for each 50 m × 50 m mesh were calculated using the regional stress and orientations of the meshed surfaces. The normalized slip tendency (${T}_{s}^{^{\prime}}$) of 0.1–1.0 was calculated for the study area, assuming conventional frictional coefficients of 0.6 ^19^ (Fig. 3A). The ${T}_{s}^{^{\prime}}$ exhibited a heterogeneous distribution with weak trends in the NE–SW or ENE–WSW directions. An area of very small (< 0.2) ${T}_{s}^{^{\prime}}$ was identified in the south where the décollement had higher dip angles. We also examined ${T}_{s}^{^{\prime}}$ with a friction coefficient of 0.4 (Fig. 3C). This smaller friction coefficient was from laboratory experiments under slow velocity conditions on hemipelagic mud core samples within the 3D seismic survey area at IODP Site C0004^20^. Although the smaller friction coefficient of 0.4 yielded larger ${T}_{s}^{^{\prime}}$ than the larger friction coefficient, the distributions of ${T}_{s}^{^{\prime}}$ obtained with different friction coefficients were consistent each other, suggesting that distribution of ${T}_{s}^{^{\prime}}$ was primarily controlled by the décollement geometry and regional stress.

The dilation tendency (${T}_{d}$) ranged from 0 to 1, and also exhibited a heterogeneous distribution with trends in the NE–SW or ENE–WSW directions, similar to the distribution of ${T}_{s}^{^{\prime}}$ (Fig. 3B). Furthermore, areas of high and low ${T}_{d}$ coincided with those of high and low ${T}_{s}^{^{\prime}}$.

We also examined ${T}_{s}^{^{\prime}}$ as one of the preferred stress states (not the optimal stress state) with the horizontal maximum stress and a stress ratio of 0.2 that are the lowest dip angle of the horizontal maximum stress and the smallest stress ratio in the variation in stress state with 0.95 of *F*^*(i*)^ (Fig. S2). The distribution of ${T}_{s}^{^{\prime}}$ also represents a NE-SW or ENE–WSW trend, although the high ${T}_{s}^{^{\prime}}$ areas are distributed oppositely in the low ${T}_{s}^{^{\prime}}$ areas with the optimal regional stress state (Fig. 3D).

## Discussion

VLFE alignments can be expressed as the trend of map scale distributions for comparison of distributions between ${T}_{s}^{^{\prime}}$, ${T}_{d}$ and VLFE, even though the locations of the VLFE have uncertainties of 10–20 km^5^. The alignments of VLFE were detected in some of the time windows (see “Methods” section). VLFE alignments are not evenly distributed but occur as clusters in the central part of the study area, such as those on April 7th, April 8th, and 0:00–7:00 on April 10th, 2016 (Fig. S3). This is a limitation of the spatial resolution of the data used to determine the VLFE locations.

**Figure 4:**
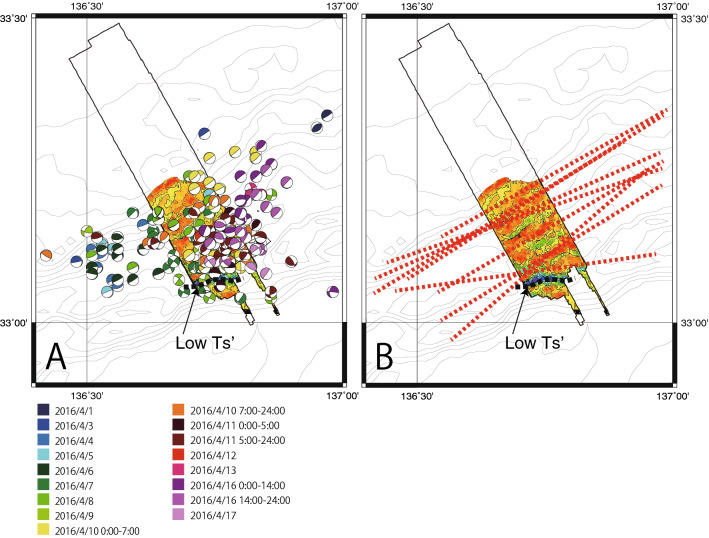
(**A**) VLFE distributions for April 2016 overlaid on the slip tendency map obtained with a friction coefficient of 0.6. Colors indicate different time windows. The black dashed curved line, which is indicated by an arrow with “Low ${T}_{s}^{^{\prime}}$”, indicates a boundary through which most of the VLFE do not migrate to the trench. (**B**) Combined alignments of VLFE in a map. Red broken lines represent alignments of VLFE identified in each time window. Identified alignments of VLFE for each time window are presented in Fig. S3.

Combining all alignments on a single map, the concentration of the aligned distributions of VLFE are recognizable (Fig. 4B). With a small number of exceptions, most of the alignments of VLFE are consistently oriented NE–SW or ENE–WSW, which is parallel to the distribution of ${T}_{s}^{^{\prime}}$ and ${T}_{d}$. Smaller segments of NW–SE aligned CMT solutions are also observed, especially from 1st to 3rd April (Fig. S3). These could be minor alignments because the length of the segment is shorter and the number of CMT solutions for the NW–SE alignment are also smaller than the NE–SW alignments. Although the locations of VLFE alignments seem to be consistent with the area of high ${T}_{s}^{^{\prime}}$ and ${T}_{d}$ with the optimal regional stress, the consistency of the locations needs to be carefully interpreted because the uncertainties of VLFE location are large. Furthermore, the alignments are located oppositely in the lower ${T}_{s}^{^{\prime}}$ area for the ${T}_{s}^{^{\prime}}$ distribution with the preferred stress state. Even so, the consistency of parallel distributions between VLFE alignments, ${T}_{s}^{^{\prime}}$ and ${T}_{d}$ can be emphasized here.

Slip along a fault is a matter of fault stability, which can be determined from the ratio of the shear stress to the normal stress as ${T}_{s}^{^{\prime}}$. Since ${T}_{s}^{^{\prime}}$ indicates the probability of reactivation of a fault, it is highly possible that VLFEs occur in regions of high ${T}_{s}^{^{\prime}}$, which is suggested by the parallel distributions between ${T}_{s}^{^{\prime}}$ and VLFE alignments.

The rate of VLFE migration from depth toward the trench is approximately 4 m/s^5^. If the VLFE are induced by fluid, this rate suggests that the décollement has a fairly high permeability. Because ${T}_{d}$ indicates the probability of fault dilation, fluid migration associated with high permeability can be examined with respect to the distribution of ${T}_{d}$. A region of high ${T}_{d}$, and thus a high probability of fault dilation, can be a conduit for fluid migration connected to deeper high-pressure fluids during dynamic events, which can enhance the slip. On the other hand, we suggest that regions of low ${T}_{s}^{^{\prime}}$ and ${T}_{d}$*,* where shear stresses are less concentrated and high-pressure fluids cannot migrate from depth, are stable areas that will act as a barrier to VLFE slip. This interpretation is weakly supported by the fact that a limited number of VLFE are observed from the regions of very low ${T}_{s}^{^{\prime}}$ and ${T}_{d}$ to the trench. The limitation is not clear in Fig. 4A, but it is weakly shown in some time windows (Fig. S3), although the uncertainty of the VLFE locations still needs to be considered.

${T}_{s}^{^{\prime}}$ and ${T}_{d}$ as determined in this study do not provide information regarding the physical properties of the rocks, and only represent the probability of fault reactivation, which is solely a geometric control on the relationship between the surface roughness and the regional stress state. Numerical simulations suggest that the roughness of the plate interface, including seamounts, can control the distribution of stress, permeability, fluid pressure, and sediment properties^10^. Those simulations thus suggest that the roughness of the plate interface can be a primary control on these properties. The results of our study also suggest that the interface geometry itself is a major control on the slip, because ${T}_{s}^{^{\prime}}$ and ${T}_{d}$ are purely geometric expressions that respond to the regional stress. Fluid migration and sediment properties can also be affected by the geometry of the plate interface controlling the ${T}_{d}$, representing another factor that may enhance slip on the décollement.

Our study demonstrates the consistency in parallel trends between VLFE alignments and distributions of ${T}_{s}^{^{\prime}}$ and ${T}_{d}$. However, these results are only for a single series of VLFE events with a duration of 17 days. Examination of other series of VLFE, with respect to décollement geometry, is required to make the geometrical control on VLFE more convincing. We should also consider the effect of geometry, not only on VLFE, but also on slow slip events or regular earthquakes.

Distributions of ${T}_{s}^{^{\prime}}$ and ${T}_{d}$ on the shallow décollement in the Nankai Trough off the Kii Peninsula, obtained using décollement geometry and the regional stress state from CMT mechanisms of VLFE, show that the trends of ${T}_{s}^{^{\prime}}$ are parallel to the aligned distributions of VLFE, suggesting that VLFE occur in areas of high ${T}_{s}^{^{\prime}}$. The distribution of ${T}_{d}$ is consistent with that of ${T}_{s}^{^{\prime}}$, which may enhance slip because the high ${T}_{d}$ areas could act as a fluid conduit during dynamic events. Because ${T}_{s}^{^{\prime}}$ and ${T}_{d}$ are derived only from the décollement geometry and regional stress, without any consideration of sediment properties, the consistency between their distributions suggests that the décollement geometry is the primary control of VLFE occurrences.

## Methods

### Constructing the décollement surface

The 3D geometry of the shallow décollement was produced using the seismic profiles from a 3D seismic reflection survey^12^. During acquisition, two air gun arrays with 75-m spacing, each totaling 50.64 L at 13.8 MPa, were alternately fired at 37.5 m shot intervals. Four 4.5-km hydrophone streamer cables with 360 receiver groups at 12.5-m spacing were towed 150 m apart. The seismic data were re-processed in 2016 and 2018 to better attenuate the noise and extract the broadband signals. The depth images were improved using pre-stack depth migration^18^. The bin size of the final version of the processed data was 12.5 m in the in-line direction (NW–SE) and 18.75 m in the cross-line direction (SW–NE). We interpreted the shallow décollement surface from the clearly visible reflections above basaltic basement within the sedimentary sequences (Fig. 1B). The depth below sea level of the interpreted décollement ranged from 4500 m at the trench axis to ~ 7000 m within the landward ~ 28 km of the trench axis (Fig. 2A). After the geometry of the décollement was interpreted, we defined a 50 m × 50 m mesh grid and determined the dip azimuth and dip angle for each grid point using the Petrel software package (Schlumberger) (Fig. 2B,C).

### Stress inversion from VLFE slip data

We used slip data on the nodal planes derived from the CMT mechanisms of the VLFE to estimate the regional stress state. Slip data consists of dip direction and dip angle of slip plane, horizontal azimuth and dip angle of slip direction, which was obtained from CMT mechanisms. Most of the CMT mechanisms consist of a pair of almost horizontal and steeply dipping nodal planes with reverse slips. The horizontal nodal planes were assumed to be actual slip planes because of the consistency with the nearly horizontal décollement.

From the slip data, we used the Hough-transformed stress inversion method^21^ to estimate the optimal stress state. This inversion method is similar to the multiple micro-fault inversion method^22^, which tries to find multiple solutions for multiple tectonic stress stages or regions using statistical analysis. The Hough transform method uses a mathematical technique to find each solution faster than other methods. The inversion method determines solutions for each selected slip data point (in this study, the number of selected slip data points, *k*, was 3) from all slip data (n = 318 from the CMT mechanisms). A solution of stress state for each calculation was obtained as the minimum summation of the misfit angle between the calculated shear directions and the observed slip directions along slip planes. Each solution was calculated repeatedly for each selected slip data point. Therefore, the number of solutions is the combination of the number of selected slip data and the number of all slip data (*nCk*), 5,309,116 in this study. The 5,309,116 solutions are plotted in 60,000 grids of stress states. Each grid indicates one stress state composed of the orientations of the principal stresses and stress ratio.

The stress ratio ($\Phi$) is defined by:

1 $$\Phi =\frac{{\sigma }_{2}-{\sigma }_{3}}{{\sigma }_{1}-{\sigma }_{3}},$$

where ${\sigma }_{1}$, ${\sigma }_{2}$, and ${\sigma }_{3}$ are the maximum, the intermediate, and the minimum principal stresses, respectively.

The normalized frequency for *i*th grid, *F*^*(i)*^ is represented by:

2 $${F}^{(i)}=\frac{{m}^{(i)}-{m}_{min}}{{m}_{max}-{m}_{min}},$$

where *m*^*(i)*^ indicates the number of solutions in the *i*th grid of stress state, *m*_*max*_ is the maximum number of solution, and *m*_*min*_ is the minimum number of the solution among all grids of stress state. The optimal solution is determined as the grid of the stress state obtaining the largest number of solutions from the total (5,309,116) possible solutions^21^, which means *F*^*(i)*^ = 1. The variation of the preferred solution, not the optimal solution, was examined over 0.95 of *F*^*(i)*^ in this study (Fig. S1)*.*

This method can separate different stress histories or regions if they exist, represented by multiple clusters of the solutions. The stress state obtained in this study was observed as a single cluster (Fig. S1), which implies that the single cluster represents the regional stress covering the VLFE area.

### Calculating slip and dilation tendencies

To calculate the slip and dilation tendencies, we used the notional magnitudes of the principal stresses^23^. Slip tendency^24^ $({T}_{s}$) is defined as:

3 $${T}_{s}= \frac{\tau }{{\sigma }_{n}},$$

where ${\sigma }_{n}$ and $\tau$ indicate the normal and shear stresses on the plane in each 50 m × 50 m mesh surface on the décollement. For known values of the principal stresses, the normal and shear stresses affecting a plane depend on the orientations of the plane^25^:

4 $${\sigma }_{n}={\sigma }_{1}{l}^{2}+{\sigma }_{2}{m}^{2}+{\sigma }_{3}{n}^{2},$$

5 $$\tau =\sqrt{{\left({\sigma }_{1}-{\sigma }_{2}\right)}^{2}{l}^{2}{m}^{2}+{\left({\sigma }_{2}-{\sigma }_{3}\right)}^{2}{m}^{2}{n}^{2}+{\left({\sigma }_{3}-{\sigma }_{1}\right)}^{2}{l}^{2}{n}^{2}},$$

where *l*, *m*, and *n* are the direction cosines of the mesh surface normal to the principal stress axes. Since the actual magnitudes of the principal stresses are unknown, the principal stresses can be rewritten notionally with the stress ratio ($\Phi$) and two unknown parameters, differential stress (*k*_1_) and the minimum principal stress (*k*_2_):

6 $${\sigma }_{1}={k}_{1}+{k}_{2},$$

7 $${\sigma }_{2}={k}_{1}\Phi +{k}_{2},$$

8 $${\sigma }_{3}={k}_{2}.$$

Assuming a frictional sliding envelope with a friction coefficient as the maximum slip tendency (${T}_{s\_max}$), the principal stresses can be constrained by:

9 $${\sigma }_{1}=\frac{{k}_{1}\left(csc\theta +1\right)}{2},$$

10 $${\sigma }_{2}={{\sigma }_{1}-k}_{1}\left(1-\Phi \right),$$

11 $${\sigma }_{3}={\sigma }_{1}-{k}_{1},$$

where θ is the angle of the frictional sliding envelope. These principal stresses are represented by notional magnitudes consistent with the constraints of the friction angle and a known stress ratio. We then substituted the notional magnitudes of the principal stresses into Eqs. (4) and (5) and then used Eq. (3) to independently calculate ${T}_{s}$ using any choice of unknown ${k}_{1}$ (Fig. S2) because ${\sigma }_{1}$, ${\sigma }_{2}$, ${\sigma }_{3}$, ${\sigma }_{n}$ and $\tau$ are all proportional to ${k}_{1}$, while ${k}_{1}$ is reduced in the calculations of ${T}_{s}$ and ${T}_{d}$. This means that the absolute value of stress magnitude is not needed, and we can use any value of ${k}_{1}$ in the calculation. The friction coefficient as the maximum slip tendency (${T}_{s\_max}$) was set as 0.6 and 0.4 (Fig. 3A,C). The slip tendency for a preferred stress state with the horizontal maximum principal stress and smaller tress ratio of 0.2 (Fig. S1B) is also examined (Fig. 3D). Finally, the ${T}_{s}$ is normalized by the maximum slip tendency (${T}_{s}^{^{\prime}}$) ranging from 0 to 1,

12 $${T}_{s}^{^{\prime}}=\frac{{T}_{s}}{{T}_{s\_max}}.$$

The dilation tendency^26^ is defined as:

13 $${T}_{d}= \frac{{\sigma }_{1}-{\sigma }_{n}}{{\sigma }_{1}-{\sigma }_{3}}.$$

${T}_{d}$ describes the tendency of fault opening. The notional magnitudes of the principal stresses were used to calculate ${T}_{d}$ in the same manner as for ${T}_{s}$ using Eqs. (9)–(11) for the principal stresses and Eq. (4) for the normal stress (Fig. 3B).

### Identification of VLFE alignments

The time series of VLFE used in this study covers the period from April 1st to April 17th, 2016^5^. This time series of VLFE distributions was divided into time windows for snapshots of VLFE distributions (Fig. 4A). The time windows were ordered by day, but some of time windows of a single day were further divided into two parts (Fig. 4A). Although the distributions of the VLFE are variable among the time windows, alignments of VLFE were clearly visible in some time windows. Most of the alignments represent NE–SW or ENE–WSW trends (Fig. 4A, Fig. S3). Some down-dip alignments in the NW–SE direction are also observed, especially during 1st to 3rd April (Fig. S3, green dashed lines). The down-dip alignments are relatively shorter in length with smaller numbers of CMT solutions. Therefore, we define the NE–SW or ENE–WSW trends of alignments as major trends of the VLFE distributions. Alignments of VLFE with relatively narrow width, isolated from others and composed of more than 6 CMT solutions (the average of the number of CMT solution in each alignment is ~ 9) were hand picked (dashed black boxes in Fig. S3). We did not include the CMT mechanisms in a cluster at the middle part of the area for the VLFE event for the identification of the alignment (VLFE clusters on April 7th, April 8th, and 0:00–7:00 on April 10th) (Fig. S3) because those VLFEs are not consistently aligned in the cluster in different time windows. The alignments were drawn as the best fitting lines of the locations of CMT mechanisms in each black box (dashed red lines in Fig. S3).

## Supplementary Information

Supplementary Legends.

Supplementary Figure S1.

Supplementary Figure S2.

Supplementary Figure S3.

## Data Availability

Derived data supporting the findings of this study are available from http://www-solid.eps.s.u-tokyo.ac.jp/~sloweq/original/Nakano2018-VLF-original.zip for VLFE CMT mechanisms and from the JAMSTEC Data Catalog (http://www.godac.jamstec.go.jp/catalog/data_catalog/e/index.html) for datasets related to the 3D seismic volume. However, the latter link to the database site is currently not working because JAMSTEC faced a security incident in March 2021 and is still blocking external accesses to the catalog for several months. We expect this will be resolved soon.
