# Supplementary material for: Décollement geometry controls on shallow very low frequency earthquakes
Source: Sci Rep. 2022 Feb 17;12:2677. doi: 10.1038/s41598-022-06645-2 (PMC8854613; doi:10.1038/s41598-022-06645-2)
Supplement: Supplementary file 1 — Supplementary Legends. [file 41598_2022_6645_MOESM1_ESM.docx]

Auxiliary Material for

**Décollement geometry controls on shallow very low frequency earthquakes**

Yoshitaka Hashimoto^1^, Shigeyuki Sato^1^, Gaku Kimura^2^, Masataka Kinoshita^3^, Ayumu Miyakawa^4^, Gregory F. Moore^5^, Masaru Nakano^6^, Kazuya Shiraishi^6^ and Yasuhiro Yamada^6^

^1^ Department of Global Environment and Disaster Prevention, Faculty of Science and Technology, Kochi University, Akebonocho 2-5-1, Kochi 780-8520, Japan

^2^ Tokyo University of Marine Science and Technology, 4-5-7 Kounan, Minato-ku, Tokyo 108-8477, Japan

^3^ Earthquake Research Institute, The University of Tokyo, 1-1-1 Yayoi, Bunkyo-ku, Tokyo 113-0032, Japan

^4^ Geological Survey of Japan, National Institute of Advanced Industrial Science and Technology, 1-1-1 Higashi, Tsukuba City, Ibaraki 305-8567, Japan

^5^ Department of Earth Sciences, University of Hawai‘i, Mānoa, 1680 East-West Road, Honolulu, HI 96822 USA

^6^ Japan Agency for Marine-Earth Science and Technology, 3173-25 Showa-machi, Kanazawa-ku, Yokohama, Kanagawa, 236-0001, Japan

**Contents of this file**

Figures S1 to S3

**Introduction**

In this study, we conducted stress inversion of slip data from CMT mechanisms of VLFE for the optimal stress state and their variations for preferred stress state, as well as calculations of normal and shear stresses on each mesh surface along the décollement corresponding to the regional stress state in a notional Mohr’s circle, and detection of alignments of VLFEs in specific time windows. The optimal and preferred solutions of the inverted stress states are represented in Figure S1 (fs01.pdf). Shear and normal stresses plotted in a notional Mohr’s circle are shown in Figure S2 (Fs02.pdf). Alignments of VLFEs in each time window are represented in Figure S3.

**Figure S1. A)** The optimal solution of the stress state, derived from VLFE slip data, shown in a lower hemisphere equal area stereonet. Black arrows indicate the VLFE slip data. Location and directions of arrows correspond to the poles of slip planes and the slip directions of the lower-side of blocks. Gray arrows indicate the calculated slip data. The triangle, diamond, and star correspond to the directions of estimated
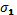
$\sigma_{1}$, $\sigma_{2}$, and $\sigma_{3}$
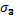
. B) Variations of preferred stress states with 0.95 of *F^(i)^* (see Method). Encircled areas with dashed lines represent $\sigma_{1}$ in the lower right and $\sigma_{3}$ in the upper left of the stereonet. Stress ratio is shown by color. One of the preferred stress states examined for the distribution of *Ts’* (Figure 4D) is pointed to by arrows.

**Figure S2.** Distributions of
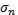
 normal stress, $\sigma_{n}$ and shear stress, $\tau$ for each 50 m x 50 m mesh surface on the décollement with the notional magnitudes of the principal stresses in a Mohr Circle. An example of normal and shear stresses on a mesh surface is located by red dashed lines at values a and b. The maximum slip tendency is represented by the slope with the frictional coefficient, µ or with tan θ.

**Figure S3.** VLFE distributions during each time window. Rectangles with broken black lines represent clusters of CMT solutions for each alignment of VLFE. Red broken lines indicate trends of alignment of VFLE parallel to the long lines of the rectangles, which is collated in a map in Fig 4B. Dashed circles indicate the area of clusters of VLFE. Wavy dashed line shows low *Ts’* and *Td* area. Green dashed lines indicate NW-SE alignments that are shorter than the NE-SW alignments.
