# Supplementary figures and images for: Décollement geometry controls on shallow very low frequency earthquakes
Source: Sci Rep. 2022 Feb 17;12:2677. doi: 10.1038/s41598-022-06645-2 (PMC8854613; doi:10.1038/s41598-022-06645-2)

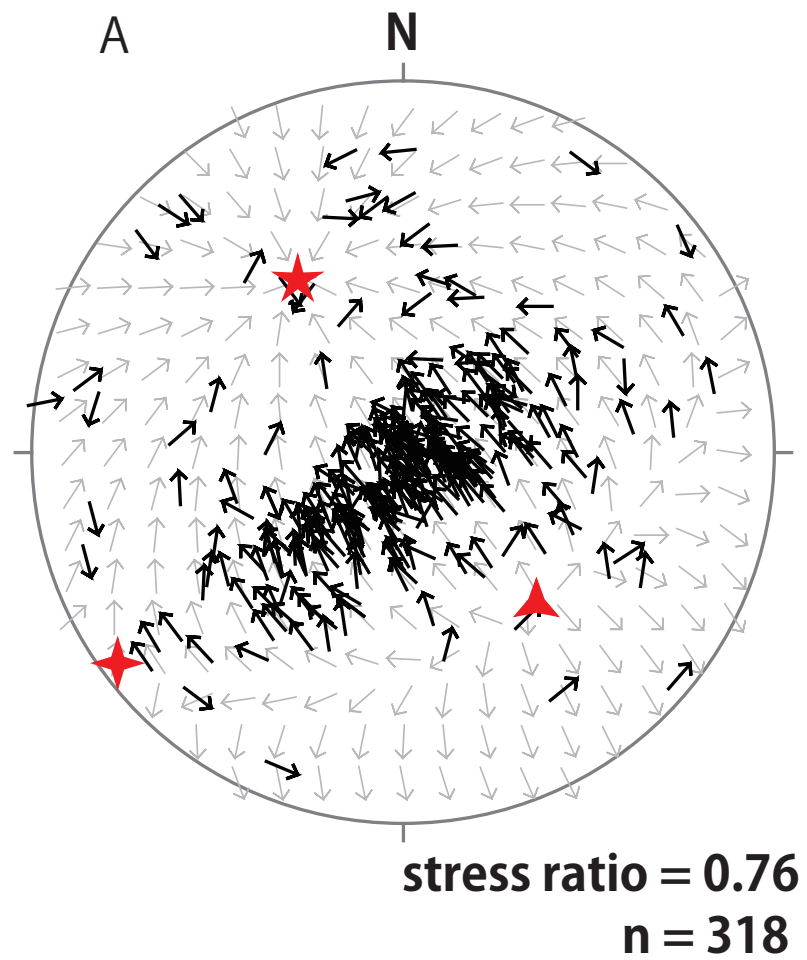

- ▲  $\sigma_1$  (139, 45)
- ◆  $\sigma_2$  (234, 5)
- ★  $\sigma_3$  (329, 45)

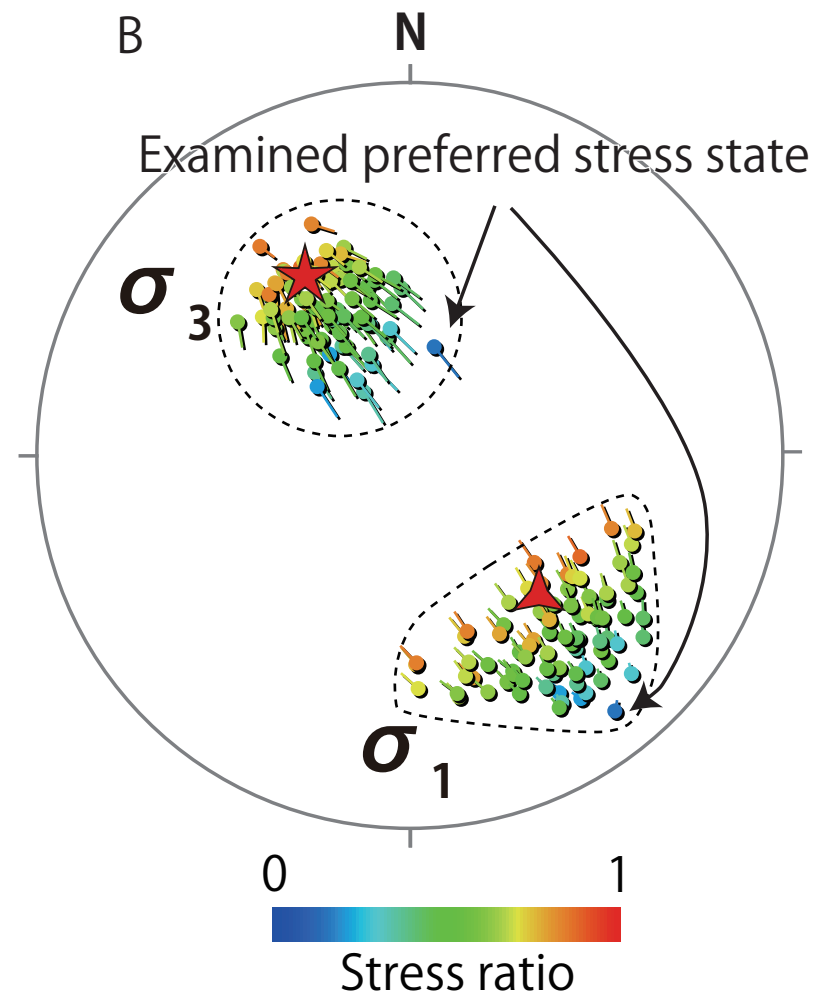

Figure S1

Supplement: Supplementary file 2 — Supplementary Figure S1. [file 41598_2022_6645_MOESM2_ESM.pdf]

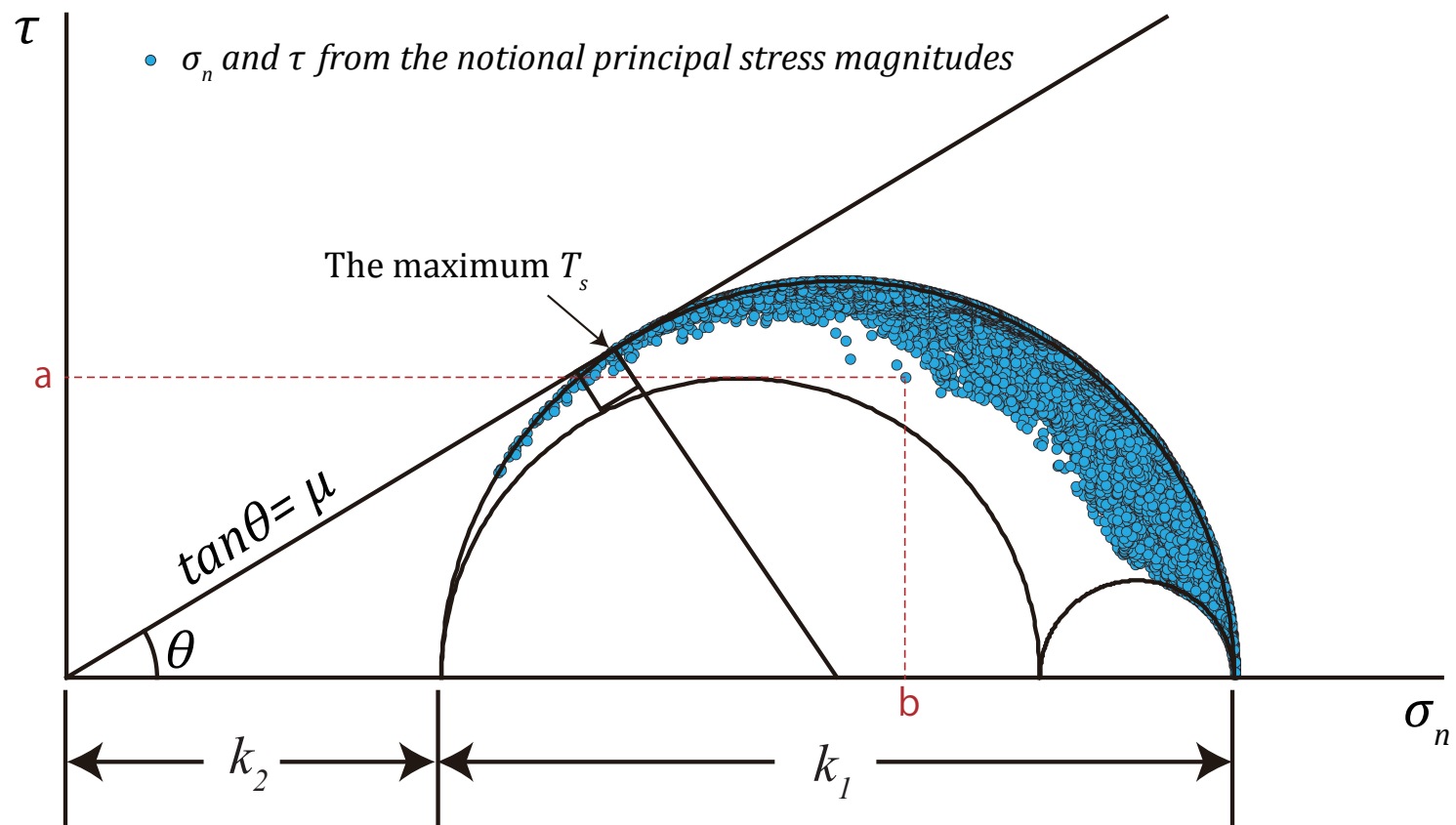

Figure S2

Supplement: Supplementary file 3 — Supplementary Figure S2. [file 41598_2022_6645_MOESM3_ESM.pdf]

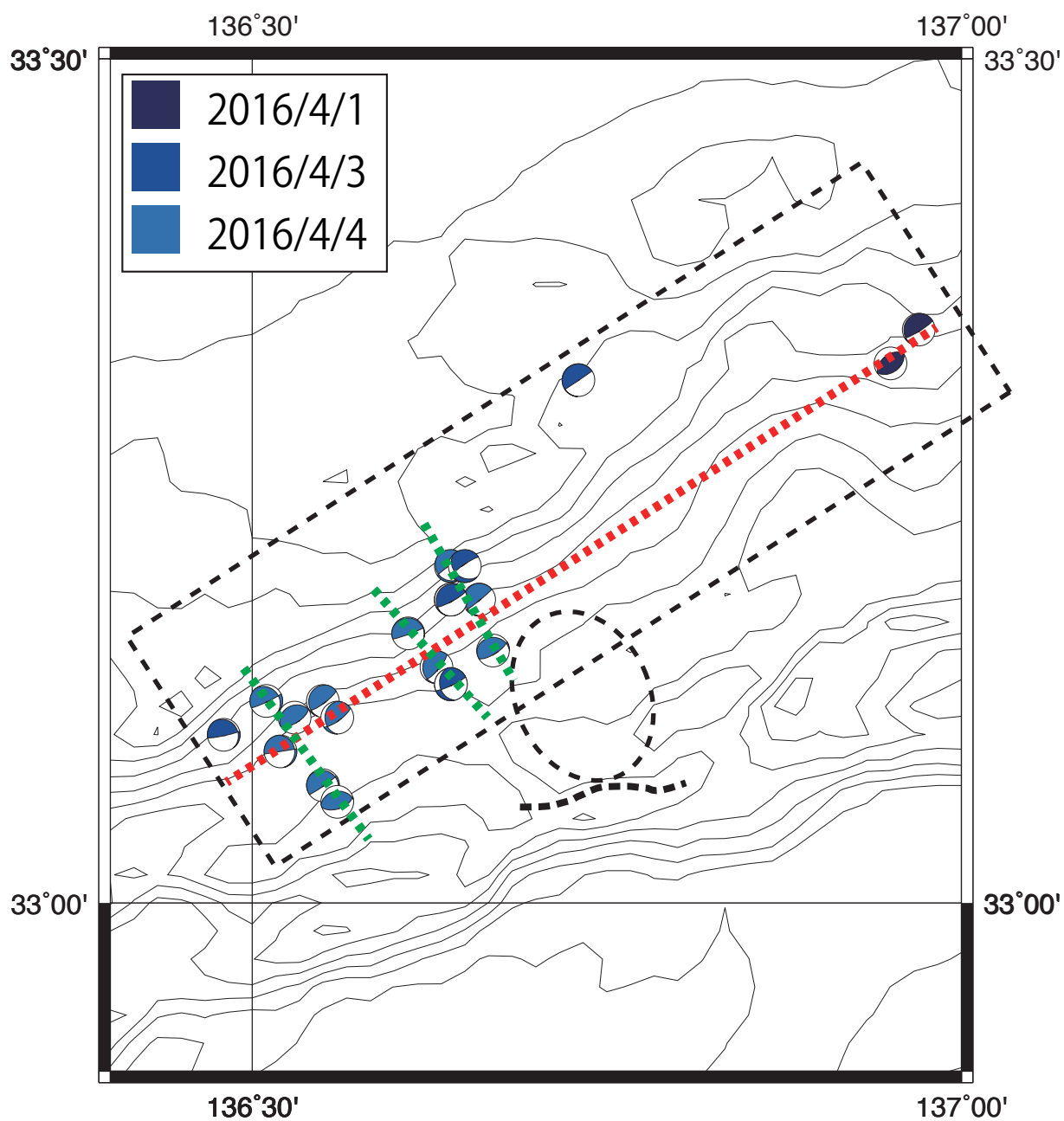

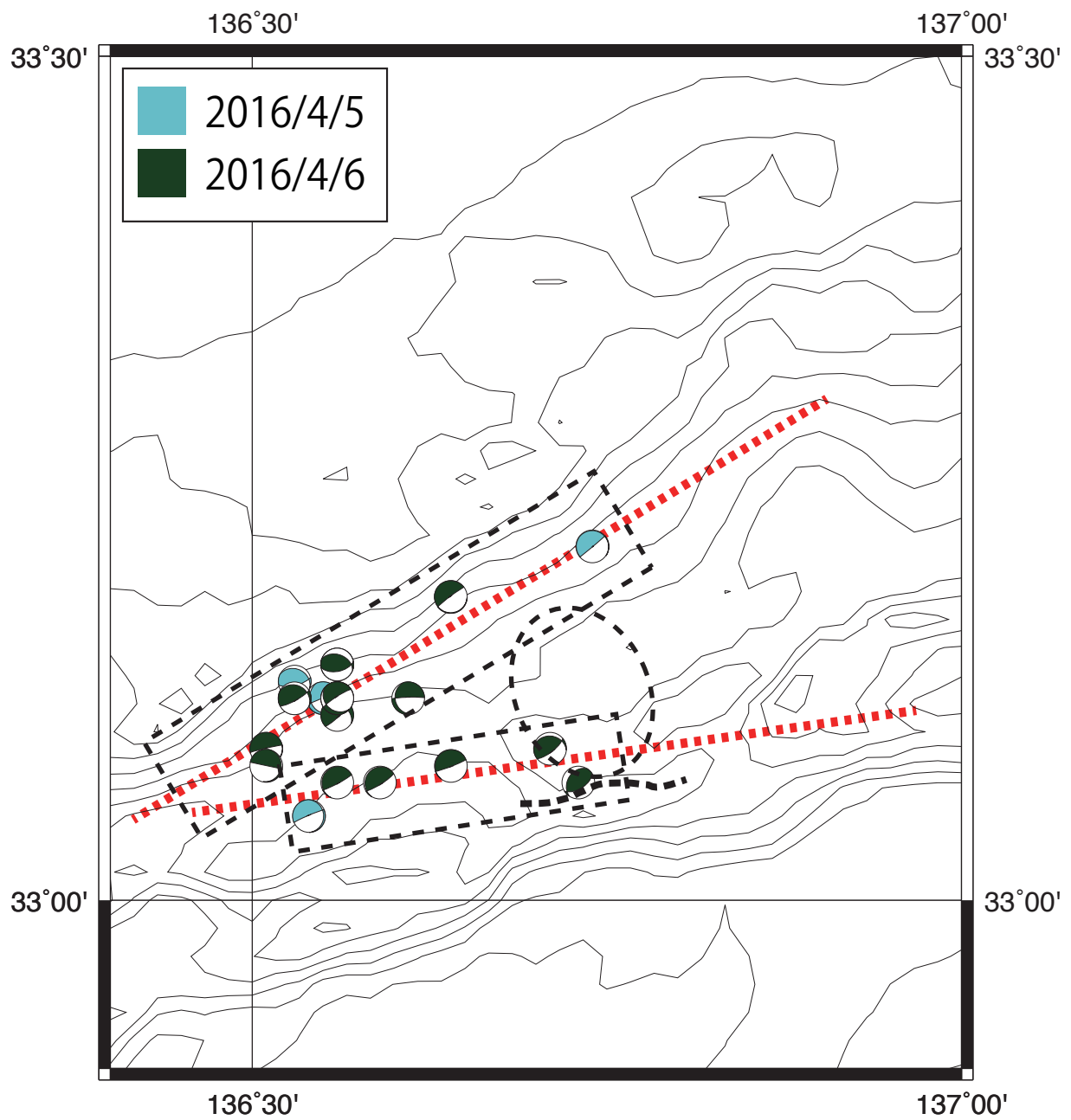

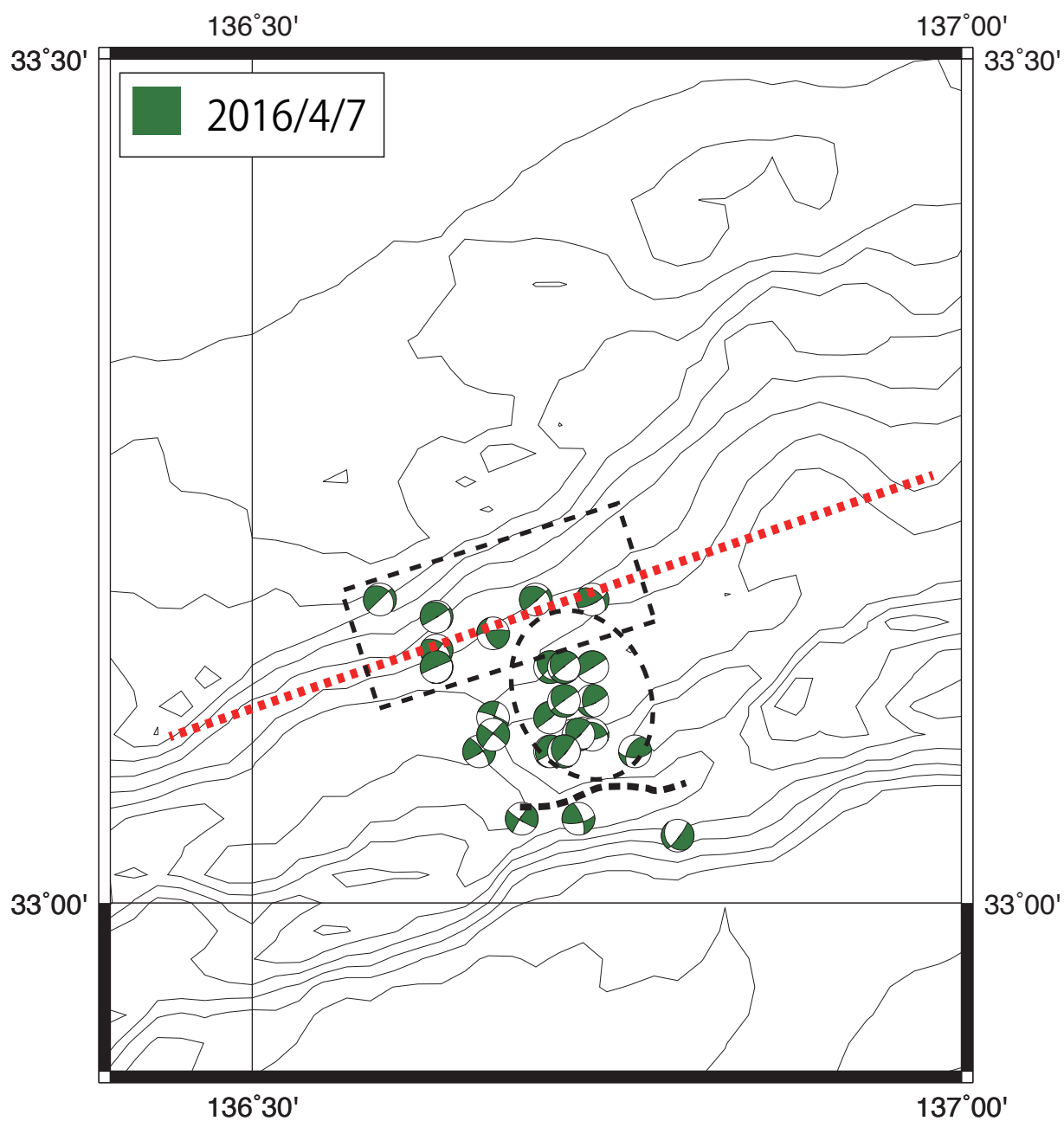

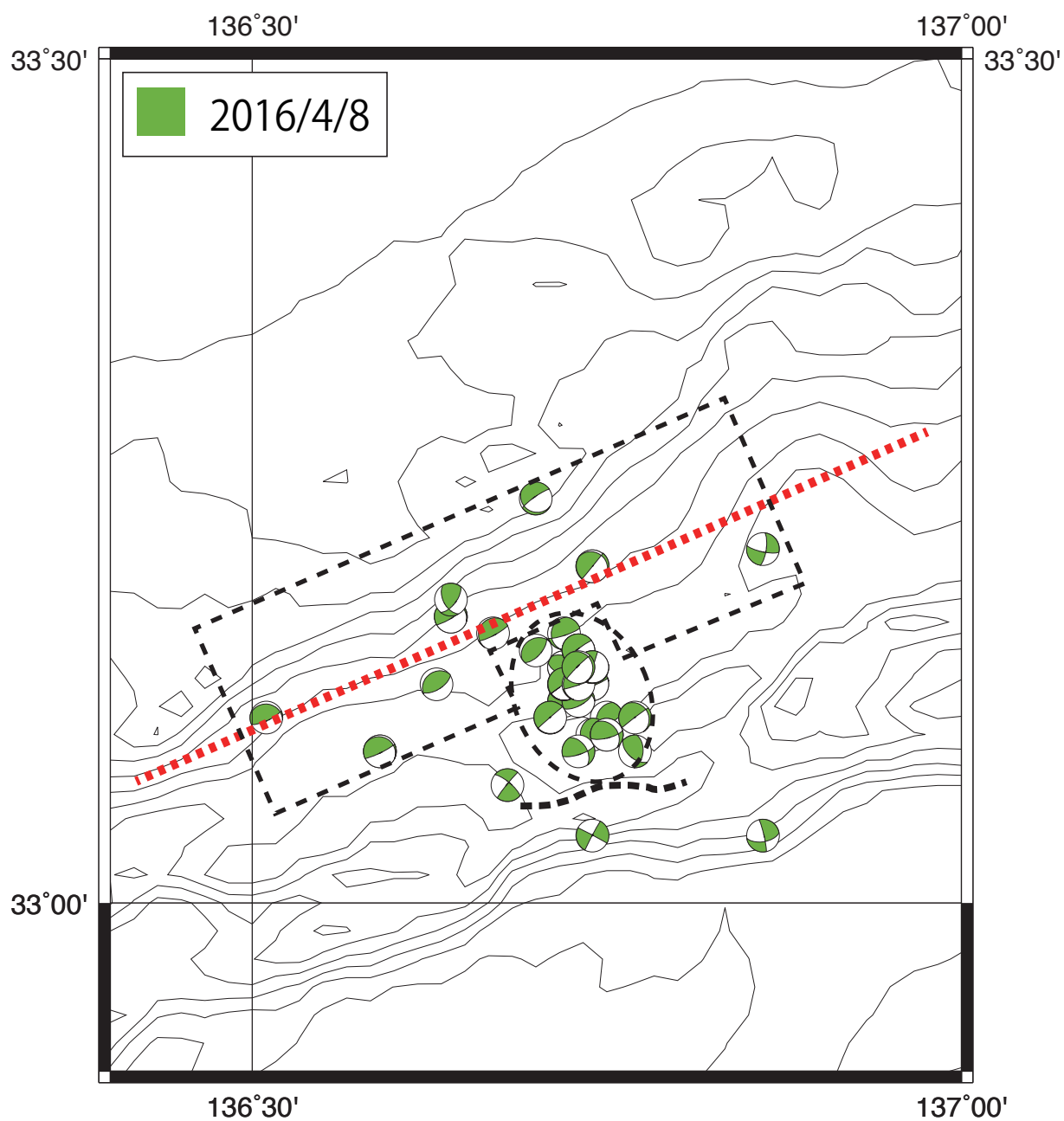

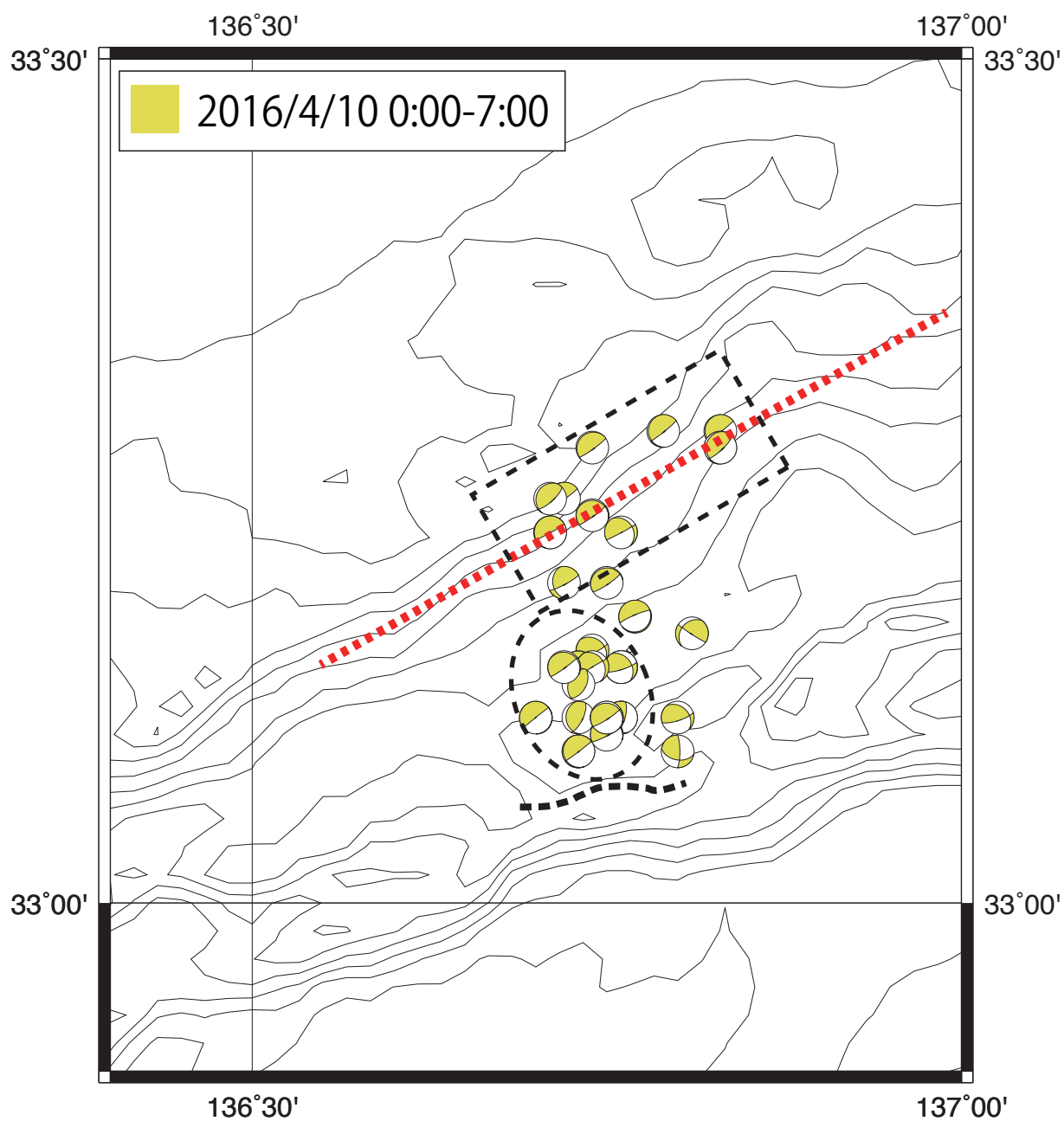

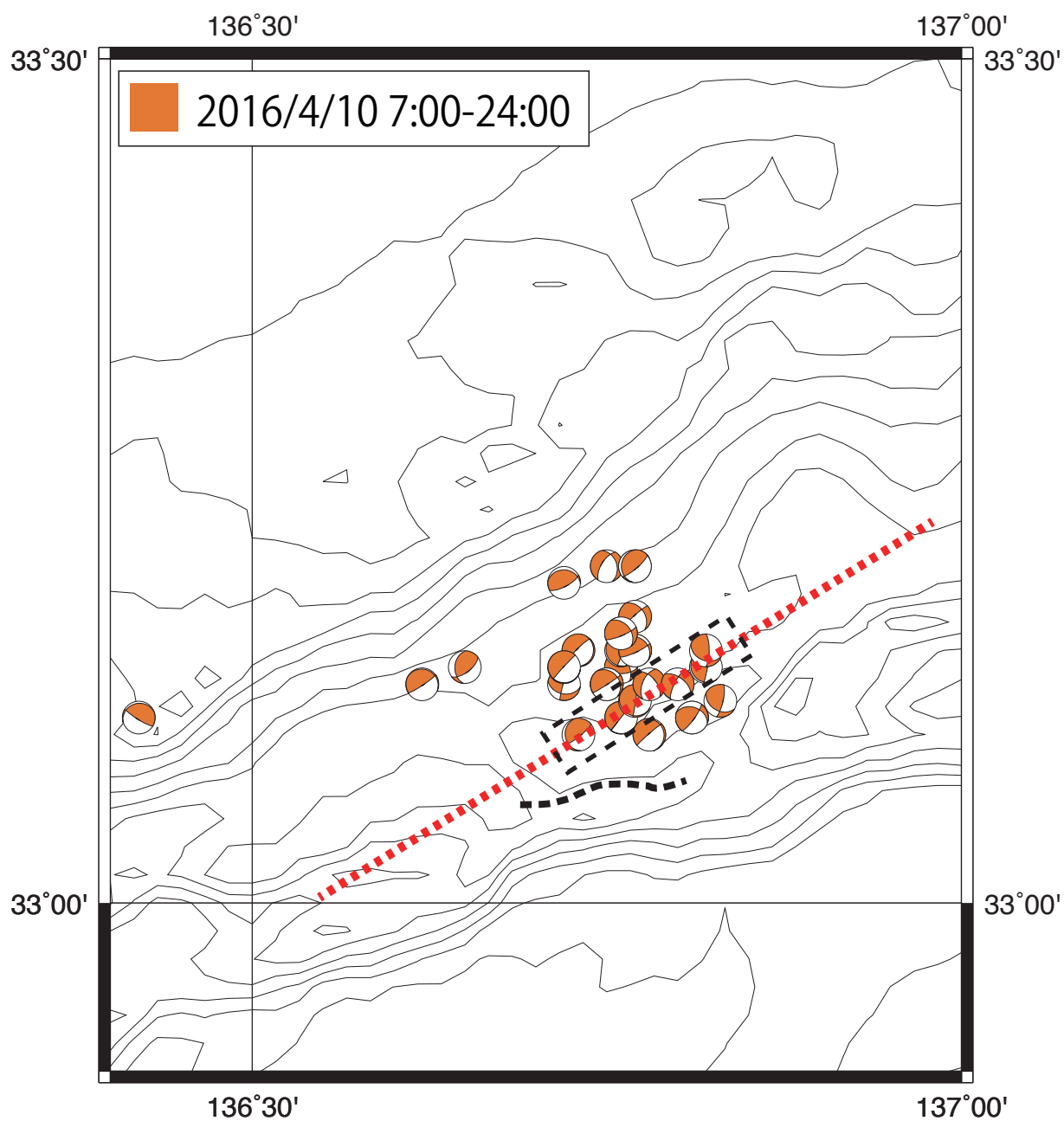

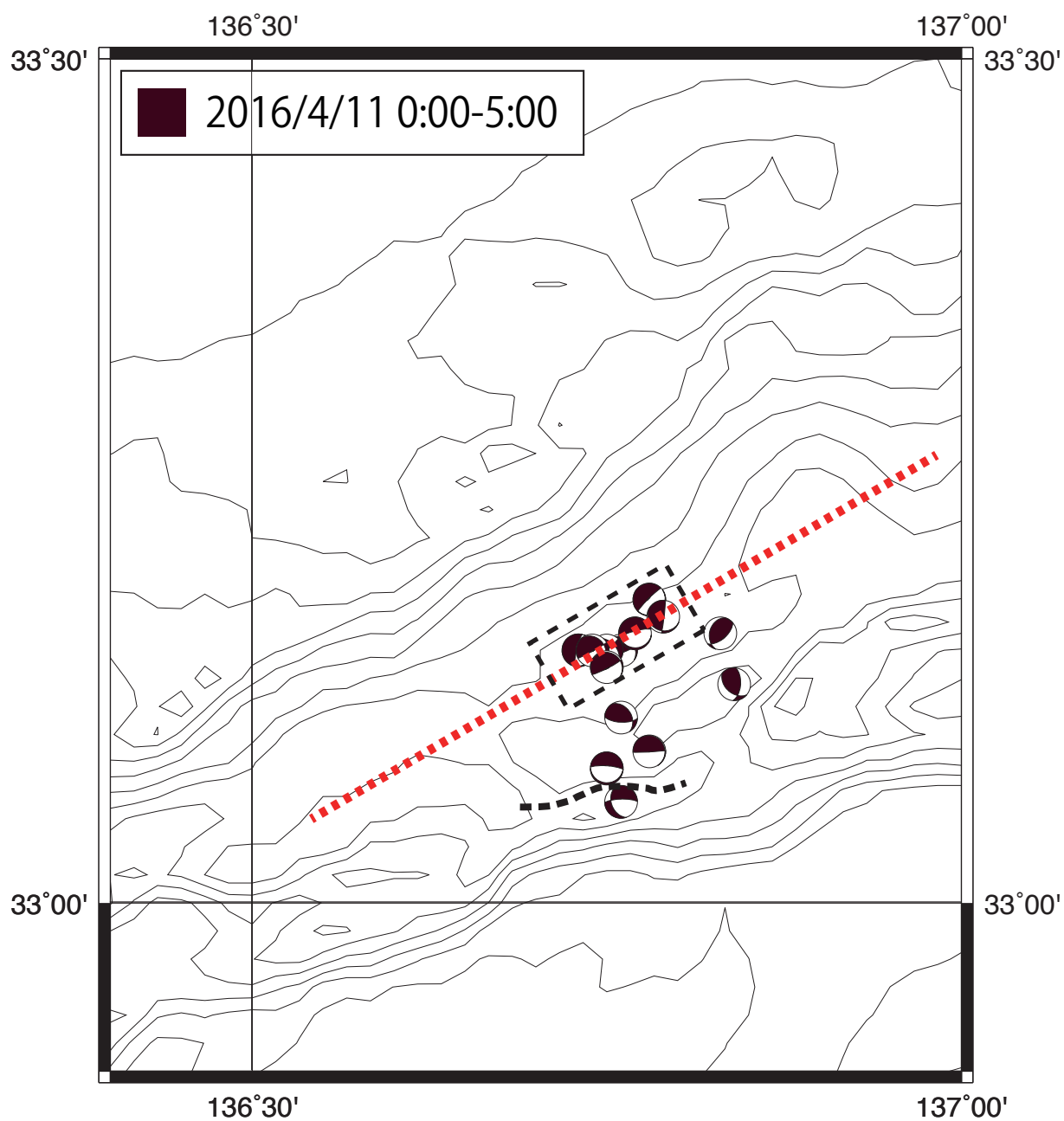

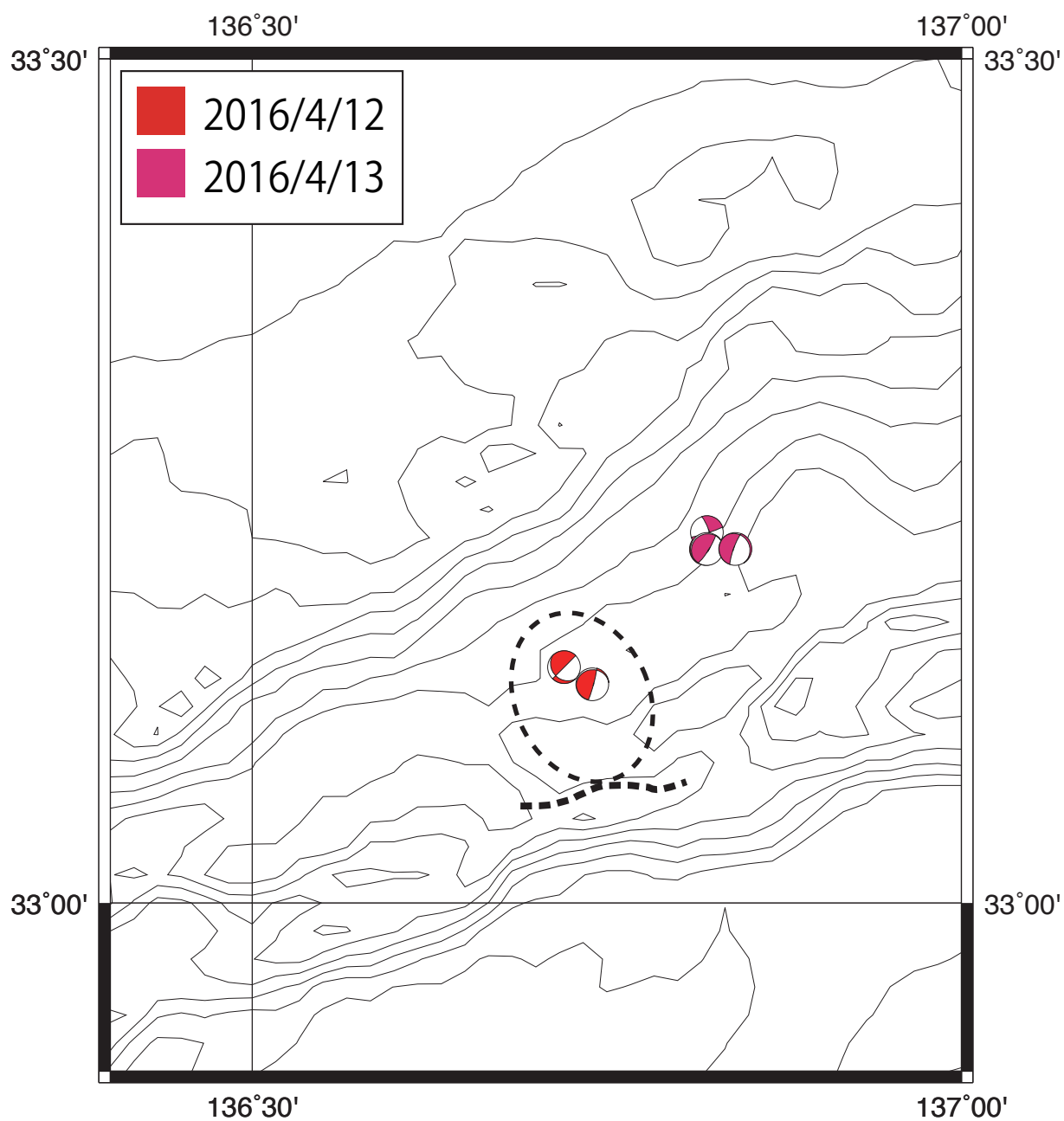

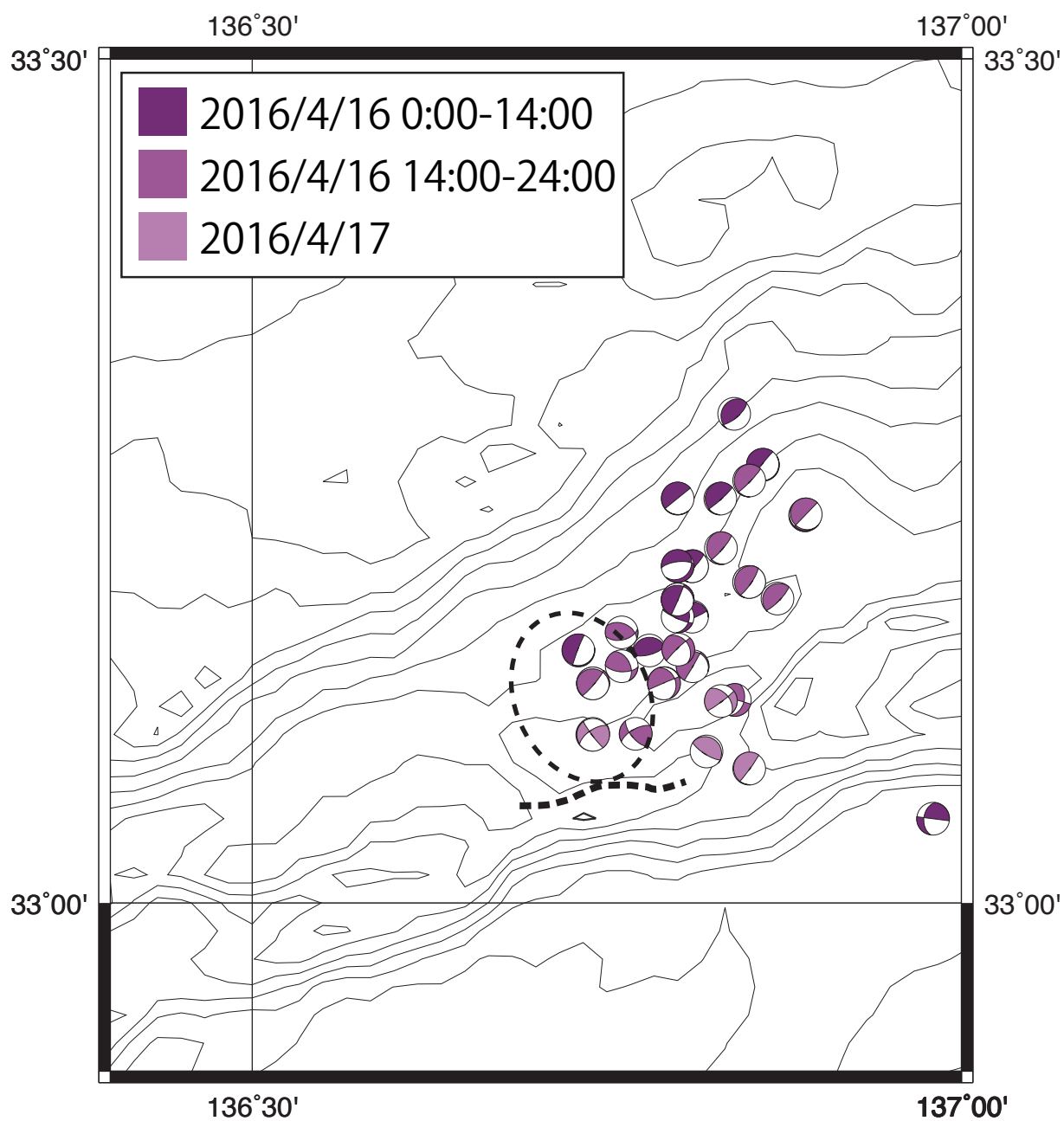

Supplement: Supplementary file 4 — Supplementary Figure S3. [file 41598_2022_6645_MOESM4_ESM.pdf]
